# 3D-QSAR modelling dataset of bioflavonoids for predicting the potential modulatory effect on P-glycoprotein activity

**DOI:** 10.1016/j.dib.2016.08.004

**Published:** 2016-08-04

**Authors:** Pathomwat Wongrattanakamon, Vannajan Sanghiran Lee, Piyarat Nimmanpipug, Supat Jiranusornkul

**Affiliations:** a Laboratory for Molecular Design and Simulation (LMDS), Department of Pharmaceutical Sciences, Faculty of Pharmacy, Chiang Mai University, Chiang Mai 50200, Thailand; b Department of Chemistry, Faculty of Science, University of Malaya, Kuala Lumpur 50603, Malaysia; c Computational Simulation and Modelling Laboratory (CSML), Department of Chemistry, Faculty of Science, Chiang Mai University, Chiang Mai 50200, Thailand

**Keywords:** Molecular modelling, QSAR, Multiple linear regression, P-glycoprotein, Flavonoids, Herb-drug interaction

## Abstract

The data is obtained from exploring the modulatory activities of bioflavonoids on P-glycoprotein function by ligand-based approaches. Multivariate Linear-QSAR models for predicting the induced/inhibitory activities of the flavonoids were created. Molecular descriptors were initially used as independent variables and a dependent variable was expressed as pFAR. The variables were then used in MLR analysis by stepwise regression calculation to build the linear QSAR data. The entire dataset consisted of 23 bioflavonoids was used as a training set. Regarding the obtained MLR QSAR model, *R* of 0.963, *R*^2^=0.927, $R_{\mathrm{adj}}^{2}=0.900$, SEE=0.197, *F*=33.849 and *q*^2^=0.927 were achieved. The true predictabilities of QSAR model were justified by evaluation with the external dataset (Table 4). The pFARs of representative flavonoids were predicted by MLR QSAR modelling. The data showed that internal and external validations may generate the same conclusion.

**Specifications Table**

**Table:** 

Subject area	*Computational Chemistry*
More specific subject area	*Quantitative Structure-Activity Relationship (QSAR) modelling*
Type of data	*Equation, tables, graphs*
How data was acquired	*In silico analysis and statistical modelling*
Data format	*Analysed*
Experimental factors	*Multivariate Linear-QSAR models for predicting the induced/inhibitory activities of the flavonoids were created. Molecular descriptors were initially used as independent variables and a dependent variable was expressed as pFAR; −log(fluorescence activity ratio).*
Experimental features	*The molecular descriptors and pFAR values were used in multiple linear regression (MLR) analysis by stepwise regression calculation to generate the model. The entire dataset consisted of 23 bioflavonoids was used as a training set.*
Data source location	*Laboratory for Molecular Design and Simulation (LMDS), Department of Pharmaceutical Sciences, Faculty of Pharmacy, Chiang Mai University, Chiang Mai, Thailand*
Data accessibility	*The data is with this article.*

**Value of the data**

• P-gp is an important clinically mediated target of herbal compounds including flavonoids in herb-drug interactions that physicians must be aware for a safe prescription.
• 3D-QSAR modelling data was constructed for predicting P-gp inhibitory activity as pFAR values of flavonoids that may allow a primary screening for healthcare providers and benefit for patients who take more than one medication.
• The model could be utilised to screen the potential herb-drug interaction risks of flavonoids and be an alternative strategy to scrutinise flavonoids which are used to recover the pharmacological outcomes of anticancers agents which are P-gp׳s substrates.

## 1 Data

The data shown here regarding a QSAR equation construction that is used to predict the induction/inhibition of P-glycoprotein modulators.

## 2 Experimental design, materials and methods

### 2.1 Dataset for analysis

The 23 flavonoids and their induced/inhibitory activities were obtained from two publications [1], [2]. The bioassay (fluorescence activity ratio; FAR at 40 µg/ml which represents P-gp induction or inhibition) values of the 23 flavonoids cover the range from 0.5 to 46.4. From the preliminary investigation using bioassay (FAR) as a dependent variable, the obtained correlation was low and increased higher in models with excessive descriptors. The FAR values were transformed becoming the corresponding pFAR (−log FAR) values, which is in the range of −1.67 to 0.3. The use of pFAR is to represent a negative value (−) as a P-gp inhibitory activity and a positive value (+) as a P-gp induced activity. Flavonoids with FAR values >1 but <10 (pFAR<0 but >−1) were regarded to be active inhibitors (weak inhibitors) of P-gp and flavonoids with FAR values >10 (pFAR<−1) were considered as potent (or strong) inhibitors [3]. A list of the flavonoid molecular structures are illustrated in Table 1 and further details on their corresponding experimental FAR and pFAR values [1], [2] are illustrated in Supplementary material.

### 2.2 Building of molecular structures

The all the two-dimensional (2D) structures of flavonoids were sketched using the ChemBioDraw Ultra. And then, the 2D structures were transformed into three-dimensional (3D) structures by using the ChemBio3D Ultra. Every hydrogen atom is regarded during the computing process for each molecule. Energy minimisation and optimisation of molecular 3D structure were also carried out utilising the ChemBio3D Ultra by MM2 forcefield.

### 2.3 Generation of numerical descriptors for the training set

The ADRIANA.Code programme (Version 2.0) was employed to compute physicochemical parameters of the molecular structures of flavonoids. This programme consists of unique combining procedures for computing molecular structure descriptors on a physicochemical basis and absolute geometric. A total of 1252 descriptors were computed utilising this programme including 8 global molecular descriptors, 88 two-dimensional autocorrelation descriptors, 96 three-dimensional autocorrelation descriptors, 1024 3D property-weighted radial distribution functions (RDF) descriptors and 36 autocorrelation of surface properties descriptors (see Table S2 [4] in Supplementary material). All calculated descriptors were standardised into the *z*-scores and then were selected as independent parameters using for pFAR prediction. Stepwise multiple linear regression method was applied to create prediction model and carried out using SPSS Statistics 17.0.

Based on the flavonoid compounds in dataset, all of these 23 compounds were used as the training set and their molecular descriptors [as standardised values (*z*-scores)] for the QSAR model construction were selected. Following the analysis method from the research of Yan et al. [5], Pearson׳s correlation coefficient (*r*) analysis merged with stepwise variant selecting manner was utilised to choose the best descriptor group for modelling. Regarding this task, molecular descriptors whose the calculated Pearson׳s correlation coefficient with the P-gp modulatory function was less than 0.1 (*r* <0.1) were not utilised.

After that by considering the pairwise correlation coefficients, if the pairwise correlation coefficient among any two descriptors was higher than 0.85, the descriptor, that had the lower correlation to the P-gp modulatory activity of a compound, one of them was eliminated. The kept descriptors were opted utilising stepwise multiple linear regression (MLR) variant selecting manner [5]. First step, every descriptor chosen with correlation analysis were ranked in a descending sequence in accordance with their correlation coefficient with activity. Second step, the descriptor which had the highest correlation coefficient with activity was utilised to create an ordinary linear regression model as an initial equation. Third step, other descriptors were subsequently admixed to the initial equation one by one. Subsequent admixing a new descriptor to the initial equation, a new equation was gained, and it was appraised with a significance test. If a significant accretion was accomplished, the admixed descriptor was kept, and if a significant accretion was not noticed, the admixed descriptor was eliminated. The procedure was reiterated till no descriptor could be admixed or eliminated [6].

### 2.4 Model validation

Many models were generated, but the best model satisfied all of the following parameters:

– The number of compounds should be 3–6 times the number of molecular descriptors used in the proposed model [7].
– *R*^2^, square of regression (>0.7) [8].
– *q*^2^, cross-validated *r*^2^ (>0.5) [8].
– SEE, standard error of estimate (smaller is better) [8].
– *F*-test, *F*-test for statistical significance of the model (higher is better, for the same set of descriptors and chemicals) [8].

To test the predictive and steadiness potentiality of the created QSAR model, the model was validated utilising internal validation. The leave-one-out (LOO, *q*^2^) manner was utilised to validate the model generated by MLR QSAR. Regarding the calculation of *q*^2^, each compound in the training dataset was consecutive moved away, the equation was refit utilising same descriptors, and the pharmacological activity of the disposed compound was predicted utilising the refit equation. The *q*^2^ was calculated utilising equation;

$$q^{2}=1-\left[\sum \;{\left({\hat{y}}_{i}-y_{i}\right)}^{2}/\sum \;{\left(y_{i}-y_{mean}\right)}^{2}\right]$$

that *y*_*i*_ and *ŷ*_*i*_ are the actual and predicted activities of the *i*th compound in the training dataset, successively, and *y*_*mean*_ is the average (P-gp modulatory) activity of all compounds in the training dataset [9].

### 2.5 QSAR analysis

The 2 steps for selection of appropriate descriptors to generate a MLR model, first, 376 descriptors that were not significantly correlated with the P-gp modulatory activity (*r*<0.1) were not utilised. Second, the remaining 876 descriptors were determined the pairwise correlation coefficient and then 570 descriptors were disposed. The remaining 306 descriptors were opted utilising stepwise linear regression variable selection manner. A stepwise multiple linear regression analysis was operated utilising the remaining descriptors after selection like inputting variables. The 23 flavonoids in the training dataset were utilised to create a statistical model equation between the P-gp modulatory (pFAR) values and physicochemical descriptors. In accordance with the criteria, six physicochemical descriptors were involved in equation, which include RDF_PiChg_86, RDF_SigChg_76, 3DACorr_TotChg_9, RDF_LpEN_54, 3DACorr_PiChg_9, and RDF_SigChg_57. The intercorrelations between the six descriptors are shown in Table 2. The pFAR was represented by the ensuing equation:

$$\mathrm{pFAR}=\sum \left(C_{i}D_{i}\right)+\mathrm{Dc}$$

In the QSAR model, Dc is a constant, Di is a molecular descriptor and C is its corresponding regression coefficient in multiple linear regression equations. The corresponding regression coefficients are illustrated in the following model.

The selected model, pFAR=−0.613(RDF_PiChg_86)+0.461(RDF_SigChg_76)−0.283(3DACorr_TotChg_9)+0.207(RDF_LpEN_54)−0.284(3DACorr_PiChg_9)−0.197(RDF_SigChg_57)−0.416, was found to have values in the required range and the regression parameters and quality correlation of the significant regression equation are *N*=23, *R*=0.963, *R*^2^=0.927, $R_{adj}^{2}=0.900$, *SEE*=0.197, *F*=33.849, *p*<0.001 and internal validation (LOO method) *q*^2^=0.927 (*N* is the number of compound in the training dataset, *R* is the correlation coefficient, *R*^2^ is the coefficient of determination, $R_{adj}^{2}$ is the adjusted coefficient of determination, *SEE* is the standard error of estimate, *F* is the Fisher test and *q*^*2*^ is the cross-validated *r*^2^).

**Fig. 1:**
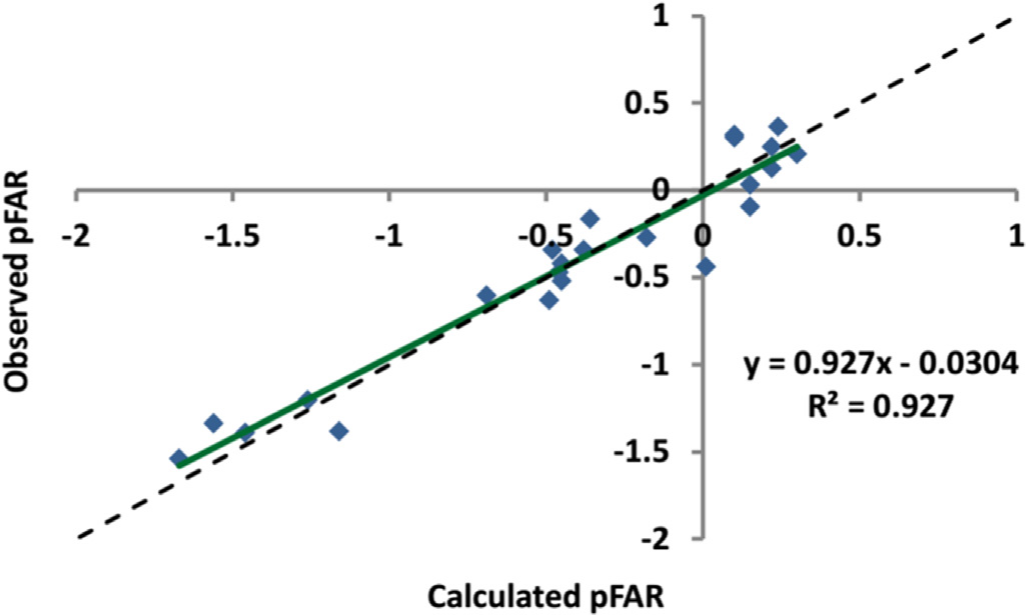
A plot of observed (experimental) versus calculated (predicted) pFAR values of the training set.

In addition, the prediction data of pFAR are listed in Table 3 and the plot of observed (experimental) versus calculated (predicted) pFAR values is shown in Fig. 1.

### 2.6 P-gp modulation prediction using the external test set of flavonoids for validation of the QSAR model

In order to evaluate the potential health risks related with herb-drug and/or food-drug interactions of some other flavonoids, the P-gp inhibitory activities of flavonoids in a dataset containing all 11 compounds (Table 4) was collected from recent the literatures [10], [11], [12], [13] which were not included in the training set and estimated using the developed QSAR model. The dataset were utilised like an external test set, which comprises all 11 active (weak) and strong inhibitors of P-gp. The values that stand for P-gp inhibitory activity of bioflavonoids from 4 literatures were converted into Inhibitory efficiency [calculated as percentage compared to a positive control (verapamil)]. The all the two-dimensional (2D) structures of 11 flavonoids were sketched using the ChemBioDraw Ultra. And then, the 2D structures were transformed into three-dimensional (3D) structures by using the ChemBio3D Ultra. All hydrogen atoms of each molecule are regarded during the computational process. Energy minimisation and optimisation of molecular 3D structure were also carried out utilising the ChemBio3D Ultra by MM2 forcefield. The ADRIANA.Code programme (Ver. 2.0) was applied to calculate physicochemical parameters of the 11 flavonoid molecules in the external test set.

All calculated descriptors were standardised into the *z*-scores and P-gp modulatory activity as pFAR values of each flavonoid were estimated using the MLR QSAR model.

The model with 6 selected molecular descriptors, which provided a good prediction operation on the external test set (Table 4), possessed high prediction accuracy that can predict the P-gp modulatory activity of 7 (from all 11) flavonoid compounds correctly including naringenin, quercetin, morin, EGCG, ECG, biochenin A and hesperidin. It could be seen that the most of the predicted flavonoid compounds showed the range of low to high predicted P-gp inhibitory activities.

## Figures and Tables

**Table 1:** Molecular structures of bioflavonoids with FAR values (in the parenthesis) of the training set. 1–21 are from Gyémant et al. [1], and 22–23 are from Martins et al. [2].

**Table 2:** Correlation matrix indicating intercorrelation among descriptors used in MLR QSAR model.

**pFAR**	RDF_Pi Chg_86	RDF_SigChg_76	3DACorr_TotChg_9	RDF_LpEN _54	3DACorr_PiChg_9	RDF_SigChg_57
RDF_Pi Chg_86	1					
RDF_SigChg_76	0.288	1				
3DACorr_TotChg_9	0.572	0.377	1			
RDF_Lp EN_54	0.529	−0.035	0.448	1		
3DACorr_PiChg_9	−0.745	−0.315	−0.299	−0.290	1	
RDF_SigChg_57	0.444	0.629	0.287	−0.033	−0.477	1

RDF_PiChg_86 is the radial distribution functions weighted by *π* charges, where *r* is in the range of 8.5–8.6 Å.

RDF_SigChg_76 is the radial distribution functions weighted by *σ* atom charges, where *r* is in the range of 7.5–7.6 Å.

3DACorr_TotChg_9 is the 3D autocorrelation weighted by total atom charges (sum of *σ*, *π* charges), where *d* is in the range of 9–10 Å.

RDF_LpEN_54 is the radial distribution functions weighted by lone pair electronegativities, where *r* is in the range of 5.3–5.4 Å.

3DACorr_PiChg_9 is the 3D autocorrelation weighted by *π* atom charges, where *d* is in the range of 9–10 Å.

RDF_SigChg_57 is the radial distribution function weighted by *σ* charge, where *r* is in the range of 5.6–5.7 Å.

**Table 3:** The observed and calculated pFAR values using the developed QSAR equation with associated residuals.

**Compound no.**	**Observed pFAR**	**Predicted pFAR**	**Residual**
1	−1.26	−1.20	−0.06
2	−1.67	−1.54	−0.13
3	−0.49	−0.63	0.14
4	−0.48	−0.34	−0.13
5	−0.45	−0.52	0.07
6	−1.46	−1.39	−0.07
7	−0.46	−0.47	0.01
8	−0.45	−0.42	−0.03
9	−0.36	−0.16	−0.20
10	−1.16	−1.38	0.22
11	−0.18	−0.27	0.09
12	−0.69	−0.60	−0.09
13	0.22	0.12	0.10
14	0.15	0.03	0.12
15	0.15	−0.09	0.25
16	0.10	0.32	−0.22
17	0.30	0.21	0.10
18	0.22	0.25	−0.03
19	0.10	0.30	−0.20
20	−0.38	−0.34	−0.04
21	−1.56	−1.34	−0.22
22	0.01	−0.44	0.45
23	0.24	0.36	−0.13

**Table 4:** Comparison between the calculated P-gp modulatory activity values (pFAR) and observed values of 11 flavonoids which exhibited a significant experimental P-gp inhibitory activity expressed by Inhibitory efficiency.

**Compound**	**Inhibitory efficiency (observed activity)**^a^	**Classification (by observed activity)**	**Calculated pFAR (Predicted activity)**	**Classification (by predicted activity)**
Naringenin	56.93	Active inhibitor	−0.39	Active inhibitor^b^
Quercetin	72.73	Active inhibitor	−0.04	Active inhibitor^b^
Morin	56.63	Active inhibitor	−0.07	Active inhibitor^c^
Silymarin	60	Active inhibitor	0.42	Inducer^c^
Epigallocatechin gallate (EGCG)	168.18	Strong inhibitor	−1.03	Strong inhibitor^d^
Epicatechin gallate (ECG)	95.45	Active inhibitor	−0.61	Active inhibitor^d^
Biochenin A	198.04	Strong inhibitor	−1.30	Strong inhibitor^b^
Hesperidin	164.41	Strong inhibitor	−1.32	Strong inhibitor^e^
Demethylnobiletin	87.43	Active inhibitor	−1.13	Strong inhibitor^e^
5HHMF	65.47	Active inhibitor	0.44	Inducer^e^
Nobiletin	45.71	Active inhibitor	1.58	Inducer^e^
Positive control (verapamil)	100	Strong inhibitor	–	–

a Inhibitory efficiency calculated as percentage compared to a positive control; verapamil.

b From Chung et al. [10].

c From Zhang and Morris [12].

d From Kitagawa et al. [11].

e From El-Readi et al. [13].
