# Supplementary material for: 3D-QSAR modelling dataset of bioflavonoids for predicting the potential modulatory effect on P-glycoprotein activity
Source: Data Brief. 2016 Aug 4;9:35–42. doi: 10.1016/j.dib.2016.08.004 (PMC5011158; doi:10.1016/j.dib.2016.08.004)
Supplement: Supplementary file 1 — Supplementary material [file mmc1.doc]

**Support information**

**Table s1**

Molecular structures of bioflavonoids with P-gp induced/inhibitory activities represented by accumulation of rhodamine 123 inside the human mdr1 gene-transfected mouse lymphoma cell line and degree of modulation represented by the Fluorescence Activity Ratio (FAR).

| **Compound no.** | **Compound name** | **FAR^a^** | **pFAR^b^** |
| --- | --- | --- | --- |
| 1. | Formononetin | 18.3 | -1.262 |
| 2. | Amorphigenin | 46.4 | -1.667 |
| 3. | Afrormosin | 3.1 | -0.491 |
| 4. | 6a,12a-Dehydroamorphigenin | 3.0 | -0.477 |
| 5. | (+)-12-Hydroxyamorphigenin (Dabinol) | 2.8 | -0.447 |
| 6. | Rotenone | 28.6 | -1.456 |
| 7. | Catechin | 2.9 | -0.462 |
| 8. | Neohesperidin | 2.8 | -0.447 |
| 9. | Naringin | 2.3 | -0.362 |
| 10. | Chrysin | 14.6 | -1.164 |
| 11. | Robinin | 1.5 | -0.176 |
| 12. | Floretin (Phloretin) | 4.9 | -0.690 |
| 13. | Floridzin (Phlorhizin) | 0.6 | 0.222 |
| 14. | Robinetin | 0.7 | 0.155 |
| 15. | Dihydrorobinetin | 0.7 | 0.155 |
| 16. | Kaempferol | 0.8 | 0.097 |
| 17. | Dihydrofisetin (fustin) | 0.5 | 0.301 |
| 18. | Dihydroquercetin | 0.6 | 0.222 |
| 19. | Sakuranin | 0.8 | 0.097 |
| 20. | Sakuranetin | 2.4 | -0.380 |
| 21. | Epigallocatechin | 36.1 | -1.558 |
| 22. | Epicatechin | 0.97 | 0.013 |
| 23. | Procyanidin B5 | 0.58 | 0.237 |
| ^a^ Average=7.65, Max.=46.4, Min.=0.5, Std.dev.=12.722, Sum=175.95. | | | |
| ^b^ Average=-0.415, Max.=0.301, Min.=-1.667, Std.dev.=0.621, Sum=-9.54. | | | |
|  | | | |

**Table s2**

Summary of 35 categories comprising 1252 molecular descriptors calculated using ADRIANA.Code.

| **Type of calculation** | **Property** | **Acronym** | **Number** |
| --- | --- | --- | --- |
| Global molecular descriptors | molecular weight of compound | Weight | 1 |
|  | number of hydrogen bonding acceptors | HDon | 1 |
|  | number of hydrogen bonding donors | HAcc | 1 |
|  | octanol/water partition coefficient in [log units] | XlogP | 1 |
|  | topological polar surface area in [Å^2^] | TPSA | 1 |
|  | mean molecular polarizability in [Å^3^] | Polariz | 1 |
|  | dipole moment in [Debye] | Dipol | 1 |
|  | solubility of the molecule in water in [log units] | LogS | 1 |
| 2D autocorrelation | atom identities | 2DA_Ident | 11 |
|  | *σ* atom charges | 2DA_SigChg | 11 |
|  | *π* atom charges | 2DA_PiChg | 11 |
|  | total charges | 2DA_TotChg | 11 |
|  | *σ* atom electronegativities | 2DA_SigEN | 11 |
|  | *π* atom electronegativities | 2DA_PiEN | 11 |
|  | lone pair electronegativities | 2DA_LpEN | 11 |
|  | effective atom polarizabilities | 2DA_Polariz | 11 |
| 3D autocorrelation | atom identities | 3DA_Ident | 12 |
|  | *σ* atom charges | 3DA_SigChg | 12 |
|  | *π* atom charges | 3DA_PiChg | 12 |
|  | total charges | 3DA_TotChg | 12 |
|  | *σ* atom electronegativities | 3DA_SigEN | 12 |
|  | *π* atom electronegativities | 3DA_PiEN | 12 |
|  | lone pair electronegativities | 3DA_LpEN | 12 |
|  | effective atom polarizabilities | 3DA_Polariz | 12 |
| Radial distribution function | atom identities | RDF_Ident | 128 |
|  | *σ* atom charges | RDF_SigChg | 128 |
|  | *π* atom charges | RDF_PiChg | 128 |
|  | total charges | RDF_TotChg | 128 |
|  | *σ* atom electronegativities | RDF_SigEN | 128 |
|  | *π* atom electronegativities | RDF_PiEN | 128 |
|  | lone pair electronegativities | RDF_LpEN | 128 |
|  | effective atom polarizabilities | RDF_Polariz | 128 |
| Surface autocorrelation | molecular electrostatic potential | Surf_ESP | 12 |
|  | hydrogen bonding potential | Surf_HBP | 12 |
|  | hydrophobicity potential | Surf_HPP | 12 |
| Total |  |  | 1252 |
